# Fertility treatment and risk of cerebral palsy: has the association changed in Australia?

**DOI:** 10.1093/humrep/deag076

**Published:** 2026-05-24

**Authors:** Michele Hansen, Anna Kemp-Casey, Sarah McIntyre, Nadia Badawi, Nicole Marie Kiss, Roger J Hart, Elizabeth Milne, Carol Bower, Shona Goldsmith

**Affiliations:** The Kids Research Institute Australia, UWA Centre for Child Health Research, University of Western Australia, Perth, Western Australia, Australia; Quality Use of Medicines and Pharmacy Research Centre, University of South Australia, Adelaide, South Australia, Australia; Cerebral Palsy Alliance Research Institute, The University of Sydney, Sydney, New South Wales, Australia; Cerebral Palsy Alliance Research Institute, The University of Sydney, Sydney, New South Wales, Australia; Grace Centre for Newborn Care, Children’s Hospital at Westmead, Sydney, New South Wales, Australia; CP Quest, Cerebral Palsy Alliance, Chance’s Clothes Project, Cowra, New South Wales, Australia; Division of Obstetrics and Gynaecology, Medical School, The University of Western Australia, Perth, Western Australia, Australia; Fertility Specialists of Western Australia, part of City Fertility Australia, Perth, Australia; The Kids Research Institute Australia, UWA Centre for Child Health Research, University of Western Australia, Perth, Western Australia, Australia; The Kids Research Institute Australia, UWA Centre for Child Health Research, University of Western Australia, Perth, Western Australia, Australia; Cerebral Palsy Alliance Research Institute, The University of Sydney, Sydney, New South Wales, Australia

**Keywords:** cerebral palsy, ART, ovulation induction, subfertility, single embryo transfer

## Abstract

**STUDY QUESTION:**

What is the association between treated (ART, ovulation induction (OI)) and untreated subfertility and risk of cerebral palsy (CP)?

**SUMMARY ANSWER:**

There is no increased risk of CP in births to subfertile untreated women; the risk of CP has declined in ART conceptions (IVF, ICSI) and is greatest in births conceived using OI medications as a sole therapy.

**WHAT IS KNOWN ALREADY:**

Nordic data suggest a decline in risk of CP for ART births over time with the shift to single embryo transfer (SET), but there are no recent data from other countries and no Australian data for births after 2002. Few studies have examined the risk of CP in births conceived after OI or untreated subfertility; these comparison groups may assist in separating ART treatment effects from potential effects of subfertility itself.

**STUDY DESIGN, SIZE, DURATION:**

Retrospective cohort study using linked population-based data for women with a live birth (LB) in Western Australia from 2003 to 2014, corresponding to 10 126 ART births (3.1%), 4102 births after OI (1.2%), 11 554 births to subfertile untreated women (3.5%), and 305 508 (92.2%) births to fertile women conceiving naturally.

**PARTICIPANTS/MATERIALS, SETTING, METHODS:**

Statutory ART, birth, hospital, CP, and pharmacy data were linked to identify four ‘conception groups’ (ART, OI, subfertile untreated, and fertile natural conceptions), outcome data, and potential confounders. CP descriptions were verified at 5 years of age. Birth prevalence of CP was calculated for each conception group per 1000 LB. Poisson regression with robust standard errors was used to estimate adjusted risk ratios (aRR) for CP with adjustment for maternal demographic characteristics, pre-existing conditions, and adverse obstetric history (with 95% CI). Births to fertile women were the reference group. Analyses included all children and singletons and twins separately. Weinberg’s differential rule was used to estimate the rate of monozygotic twinning across our four conception groups and, for ART births, by length of embryo culture.

**MAIN RESULTS AND THE ROLE OF CHANCE:**

CP was diagnosed in 29 ART children (2.9/1000 LB; 95% CI 1.92–4.11), 16 children born after OI (3.9/1000 LB; 95% CI 2.23–6.33), 23 children in the subfertile untreated group (2.0/1000 LB; 95% CI 1.26–2.99), and 610 in the fertile group (2.0/1000 LB; 95% CI 1.84–2.16). When stratified by plurality and prematurity, risk was increased only for ART twins (aRR 2.8, 95% CI 1.42–5.39) born preterm (<37 weeks) and OI singletons (aRR 2.9, 95% CI 1.41–5.84) born preterm. Twins after SET comprised an increasing proportion of all ART twins over time, and of all ART twins with CP. Most twins after SET were same-sex twins (90.7%) and we estimated that 81% were monozygous, with the majority conceived after the transfer of a single blastocyst. The monozygotic twinning rate was higher after blastocyst-SET compared with cleavage-SET (prevalence ratio 1.7 (95% CI 1.07–2.73)).

**LIMITATIONS, REASONS FOR CAUTION:**

Small numbers of children with CP in subfertile groups prevented more detailed analyses of pregnancy complications and ART cycle characteristics. More recent data from larger populations are required to confirm whether risk of CP is increased following blastocyst transfer, in twins after SET, and following OI as a sole therapy. As zygosity data were not available, Weinberg’s differential rule was used to estimate the rate of monozygotic twinning and may have led to some misclassification.

**WIDER IMPLICATIONS OF THE FINDINGS:**

Prevalence of CP following ART has declined in Western Australia, as in the Nordic countries. Avoiding multiple embryo transfer remains important. Monozygotic twinning after SET may increase the risk of CP for ART twins and is more common after blastocyst compared with cleavage-stage transfer. Embryo culture, manipulation, and freezing strategies to reduce monozygotic twinning should be explored. Data about OI (as a sole therapy) are not routinely collected in most countries, but OI-conceived births had the highest risk of CP and may warrant closer scrutiny. Pre-treatment counselling should continue to promote SET while incorporating the small risk of embryo splitting; risk of multiple birth should be discussed with those planning to use OI.

**STUDY FUNDING/COMPETING INTEREST(S):**

This work was supported by the Australian National Health and Medical Research Council, grant number 1086530 (to M.H.) and The Research Foundation of Cerebral Palsy Alliance, grant number 06221 (to M.H.). The sponsors had no role in the design or conduct of the study, or the decision to submit for publication.

Professor Hart is the Medical Director of Fertility Specialists of Western Australia and National Medical Director of City Fertility Clinic, a shareholder in CHA SMG; he has received accommodation support from Merck to attend ESHRE and has received travel and accommodation support, as well as educational sponsorship from MSD, Merck-Serono, Origio, Igenomix, and Ferring Pharmaceuticals. He has also received personal fees for presenting a Merck webinar. There are no other conflicts of interest to declare.

**TRIAL REGISTRATION NUMBER:**

N/A.

## Introduction

Cerebral palsy (CP) is an umbrella term for a group of disorders of movement and posture caused by non-progressive disturbances in the developing brain–heterogeneous, both in aetiology and clinical presentation (Dan *et al.*, 2026). There is global variation in the birth prevalence of pre/perinatal CP (brain injury prior to 28 days of life), ranging from 1.5 per 1000 live births (LB) in high-income countries to as high as 3.4 per 1000 in low- and middle-income countries (McIntyre *et al.*, 2022). Declines in the prevalence of pre/perinatal CP have been reported over time in high-income countries (Sellier *et al.*, 2016; Touyama *et al.*, 2016; Galea *et al.*, 2019), including in multiple births (Sellier *et al.*, 2021) and are thought to reflect improvements in maternal and perinatal care (McIntyre *et al.*, 2022; Smithers-Sheedy *et al.*, 2024). Contemporary Australian data (for births 2015–2016) suggest that the prevalence of pre/perinatal CP currently sits at 1.4/1000 LB (Australian Cerebral Palsy Register, 2025).

Risk factors contributing to pre/perinatal CP may play a role before or during conception, during pregnancy and/or in the perinatal/neonatal period. Over the last 20+ years, several studies have investigated whether ART treatment (standard IVF or ICSI and the transfer of fresh or frozen-thawed embryos) or ovulation induction (OI) treatments increase the risk of CP. While there is no evidence that OI or ART exert direct neurotoxic effects; several indirect pathways may link these treatments with CP (Carlsen *et al.*, 2023). Both treatments increase the likelihood of multiple gestations—through multiple embryo transfer in ART and multifollicular development in OI—which substantially elevate the risk of preterm birth. Preterm birth is a major determinant of CP, mediated in part through increased susceptibility to neonatal brain injury, including intraventricular haemorrhage and periventricular leukomalacia (Badawi *et al.*, 2021). Even among singletons, ART and OI are associated with higher rates of preterm birth (Wang *et al.*, 2022; Hansen *et al.*, 2024). Vanishing twins have also been proposed as a contributory pathway increasing risk of preterm birth, foetal growth restriction, and CP in the singleton survivor; greater risks being associated with loss at later gestational ages (Pinborg *et al.*, 2005; Kamath *et al.*, 2018; Zhu *et al.*, 2020b).

ART has also been associated with an increased risk of birth defects (Hansen *et al.*, 2013) and ART pregnancies experience more placental problems and hypertensive disorders (Stern *et al.*, 2020; Hansen *et al.*, 2024) that may contribute to risk of CP (Trønnes *et al.*, 2014; Goldsmith *et al.*, 2021; Ebbing *et al.*, 2023; Razaz *et al.*, 2023; Abe and Arima, 2025). Risks associated with underlying subfertility are also plausible, as conditions such as polycystic ovary syndrome (PCOS), endometriosis, advanced maternal age, and autoimmune or metabolic disorders may all contribute to risk of CP through similar pathways of placental dysfunction, foetal growth restriction, and preterm birth (Salem Yaniv *et al.*, 2011; Schneider *et al.*, 2018; Strøm *et al.*, 2021; Teede *et al.*, 2023).

Recent meta-analyses combining data from studies comparing risk of CP in ART versus non-ART births have suggested a 2-fold increased risk (Wang *et al.*, 2021; Cavero-Ibiricu *et al.*, 2024). When risks for singletons were examined separately, the pooled odds ratio was 1.48 (95% CI 1.23–1.79) while for multiples it was 1.05 (95% CI 0.93–1.18) (Cavero-Ibiricu *et al.*, 2024). Our previous study of Western Australian births between 1994 and 2002 also identified that the prevalence of CP was more than doubled in ART-conceived children, with risk primarily mediated by the increased proportion of multiple births and babies born preterm (Goldsmith *et al.*, 2018). Increased risks of CP have also been reported following the use of OI medications as a sole therapy (Hvidtjorn *et al.*, 2010; Klemetti *et al.*, 2010), however, results are conflicting as to whether any risks remain following stratification by plurality.

Modern ART practice has shifted to reduce obstetric complications through single embryo transfer (SET) policies, advances in culture media, improved embryo cryopreservation techniques, and better ovarian stimulation protocols resulting in fewer multiples and babies born preterm (Henningsen *et al.*, 2015; Berntsen *et al.*, 2019). Only 6% of ART births were conceived after the transfer of a single embryo in Western Australia from 1994 to 2002 (Goldsmith *et al.*, 2018), whereas SET was used in 95% of treatment cycles in Australia by 2023 (Kotevski *et al.*, 2025). This voluntary shift to SET was encouraged by supportive public funding of ART through Medicare, Australia’s universal health insurance scheme; and the Fertility Society of Australia’s Reproductive Technology Accreditation Committee guidelines recommending SET as preferred clinical practice, particularly for younger women and first cycles, from 2005 (Chambers *et al.*, 2013). The shift to SET was accompanied by a greater use of frozen-thawed versus fresh embryo transfer and a very rapid rise in blastocyst-stage embryo transfer over time. There were no births after blastocyst transfer in our previous study, but by 2023, blastocyst rather than cleavage stage embryos were transferred in 93% of embryo transfer cycles in Australia (Kotevski *et al.*, 2025). The Nordic countries have shown narrowing differences in CP risk between ART and non-ART births over time, coinciding with increased use of SET and a decline in multiple birth rates (Spangmose *et al.*, 2021; Carlsen *et al.*, 2023). However, there are no data to indicate whether the association between ART and CP has changed in other countries.

Few studies have examined the risk of CP in subfertile couples who conceive naturally (Zhu *et al.*, 2010). Subfertile and OI comparison groups can assist in separating ART treatment effects from potential effects of subfertility itself (Hansen *et al.*, 2024). Previous studies suggest that when births to subfertile treated and untreated women are compared, births to women who use ART experience more complications in pregnancy and worse perinatal outcomes than births to women who use less invasive treatments, such as OI alone, and subfertile women who conceive without treatment (Berntsen *et al.*, 2019; Stern *et al.*, 2022; Wang *et al.*, 2022; Hansen *et al.*, 2024).

In this study, we identify ART, OI, subfertile untreated, and fertile natural conceptions using whole-population administrative data (Hansen *et al.*, 2024). Children with CP were identified through linkage with a long-standing CP register (Bower *et al.*, 2015). The study aims were to provide:

- more recent Australian data on the association between ART and CP following important shifts in ART clinical practice and a declining prevalence of CP in the general population (Smithers-Sheedy *et al.*, 2024);
- the first description of CP risk in births conceived using OI medications as a sole therapy in Australia;
- a comparison group of naturally conceiving women with indicators of subfertility to investigate the association between untreated subfertility and CP.

## Materials and methods

### Data sources and study population

In this retrospective cohort study we accessed unit-record data from statutory whole-population datasets: (i) the Western Australian (WA) Midwives Notification System (midwives data)—antenatal and perinatal data on all LBs ≥20 weeks gestation (or ≥400 g if gestation unknown), (ii) Hospital Morbidity Data Collection (hospital data)—clinical information about all in-patient hospital admissions including day procedures at public and private hospitals, (iii) Birth Registrations and Death Registrations, (iv) Reproductive Technology Register (ART data)—all ART treatment cycles undertaken at fertility clinics in WA, (v) WA Register of Developmental Anomalies (WARDA, birth defects, and CP data) and, (vi) Pharmaceutical Benefits Scheme (PBS) claims (pharmacy data)—an Australian Government programme that subsidises the cost of prescription medicines for most medical conditions. Pharmacy data were supplied by the Department of Human Services, and remaining datasets were linked by the WA Data Linkage System using probabilistic matching.

ART treatment in WA is regulated by the *Human Reproductive Technology Act 1991* (The Western Australian Reproductive Technology Council, 1997) and partially subsidised through Medicare, Australia’s universal health insurance scheme (Chambers *et al.*, 2013). All clinics are required by law to provide information about every ART treatment cycle undertaken since April 1993. This information is stored on the Reproductive Technology Register maintained by the WA Department of Health. Eight clinics (all private) are currently licensed to offer ART services and there are a limited number of publicly funded cycles each year, provided through a referral process to the existing private clinics.

The cohort comprised all LBs ≥20 weeks gestation recorded in the WA Midwives data between 1 April 2003 and 31 December 2014. We included births to non-Aboriginal women only. Due to Australia’s history of colonization and continued inequity throughout society, poor perinatal outcomes (Gibberd *et al.*, 2019) and CP (Blair *et al.*, 2016; Martin *et al.*, 2023) are more common among births to Aboriginal women. We observed a much greater imbalance in the proportion of Aboriginal births in the fertile natural conception group (6.5%) versus the ART group (0.5%) than for other ethnic groups in our data. The proportion of births to Aboriginal women in the fertile group was 13 times higher than in the ART group, whereas differences for other ethnic groups (Caucasian 0.9, Asian 1.3, Indian 1.4, African 4, other 1.4) were much smaller. We did not have WA Aboriginal Health Ethics Committee approval to examine CP risk separately in Aboriginal births for this study, however, the small numbers of births to Aboriginal women following ART (<50) and OI (30) would have precluded any meaningful reporting on the risk of CP following fertility treatment in this population. Also excluded, were data from children with a known post-neonatal cause (brain injury between 28 days and 2 years of age) for their CP (n = 58), and 2756 deliveries with an indicator of fertility treatment on the Midwives record (Maternal and Child Health Data Management, 2021) but no link to an ART treatment cycle or OI medication dispensing. These births were excluded as our goal was to identify subfertile women whom we could be sure had used either ART, OI, or no treatment at all for comparison with our fertile natural conception group.

### Identification of conception groups (exposure)

We identified four conception groups: (i) ART, (ii) OI, (iii) subfertile untreated, and (iv) fertile naturally conceiving as previously described (Hansen *et al.*, 2024). In brief, the ART group comprised births occurring within 120–294 days after a maternal ART cycle. The OI group were identified by the dispensing of clomiphene, human chorionic gonadotropin, letrozole, or tamoxifen within specified time periods prior to the last menstrual period (LMP). The subfertile untreated group comprised women with a history of subfertility investigation or treatment but no evidence of ART or OI in the conception period. All remaining births were classified as fertile naturally conceived (fertile).

### Covariates

Maternal characteristics, pre-existing medical conditions, and adverse obstetric history were identified through Midwives and hospital inpatient data, and paternal age from Birth Registrations. Complications of pregnancy, labour, and delivery were identified through the Midwives birth record and by ICD codes listed for hospital admissions between the LMP and date of birth.

### Outcomes

The primary outcome was risk of CP for children born following ART, OI, or untreated subfertility versus children born to fertile women. Children with CP were identified through the statutory WARDA, considered to include virtually all children with CP in WA (Bower *et al.*, 2015). Birth and clinical information are collected and updated when children reach 5 years of age to verify the diagnosis and associated impairments. Final data extraction occurred in July 2020 allowing more than 5 years of follow-up for diagnoses of CP. WARDA also records birth defects diagnosed antenatally or before the age of 6 years (Bower *et al.*, 2015). We recorded presence/absence of a major birth defect for this study.

### Statistical analysis

Descriptive statistics were used to compare parental and birth characteristics for the different conception groups and to report clinical outcomes for those children diagnosed with CP. Group differences across categorical variables were assessed using the Pearson chi-square test or Fisher’s exact test where appropriate. Continuous variables were assessed using one-way analysis of variance. A two-sided *P* < 0.05 was considered statistically significant.

We calculated the birth prevalence of CP per 1000 LB with 95% CI for each conception group. The average annual change in CP prevalence 2003–2014 was calculated by Poisson regression with an offset term, representing the natural logarithm of the population size at risk in each year of the study. This allows for an estimate of the rate of change over time after controlling for population growth. Given the small number of children with CP in our subfertile conception groups, we calculated prevalence ratios (PR) with 95% CI to describe the change in prevalence of CP across two time periods (2003–2008 and 2009–2014).

Univariate statistics (cross-tabulations with chi-square tests and analysis of variance) were used to identify variables associated with both CP and conception group. Poisson regression with robust standard errors was used to estimate risk ratios (RR) for CP among children in each of the ART, OI, and subfertile untreated groups with naturally conceived children born to fertile women. Regression models were adjusted for potential confounders in the following order: (i) baseline/demographic characteristics (baby’s year of birth (2003–2006, 2007–2010, 2011–2014), maternal age (≤28, 29–34, 35+), parity (primiparous vs multiparous), child’s sex, maternal ethnic origin (Caucasian vs other), private health insurance at birth (yes/no), marital status (married/co-habiting vs other), smoking in pregnancy (yes/no)), (ii) pre-existing maternal medical conditions (diabetes, essential hypertension, epilepsy, anxiety and/or depression, cervical surgery), and (iii) adverse obstetric history (prior preterm birth and/or stillbirth). Analyses were performed on all births and stratified by plurality and gestational age to investigate the influence of these mediating factors. Higher order multiples were included in analyses of all births but not stratified analyses because of small numbers. Additional analyses were undertaken using generalized estimating equations with an exchangeable correlation structure to account for correlation between sibships in the cohort. There was very little missing data (private health insurance status at birth, 1.1%; marital status, 0.8%), and list-wise deletion from models was considered appropriate given the large study size. Supplementary analyses were used to investigate the impact of missing data on RR estimates for CP in all infants and stratified by plurality.

We estimated the prevalence and risk of CP with increasing numbers of embryos transferred. We also estimated the prevalence of CP for children born following different ART treatment characteristics. These analyses were restricted to singleton births following the transfer of a single embryo (SET) or twin births following double embryo transfer (DET).

As zygosity data were not available, Weinberg’s differential rule was used to estimate the rate of monozygotic twinning (Fellman and Eriksson, 2006) across our four conception groups and, for ART births, by length of embryo culture. This formula assumes that the number of dizygotic twins is double the number of unlike-sex twins. The number of monozygotic twins is then estimated by subtracting the calculated dizygotic twins from the total number of twins.

Data were analysed with SPSS version 29 (IBM SPSS Statistics, IBM Corporation, Armonk, NY) and STATA version 18.0 (StataCorp, TX, USA).

### Ethics approval

This study was approved by the WA Health Central Human Research Ethics Committee (#2015_65), the WA Reproductive Technology Council, and the Australian Institute of Health and Welfare (EO2015/4/211) as well as the WARDA Community Reference Group and Cerebral Palsy Advisory Subcommittee.

## Results

**Figure 1.**
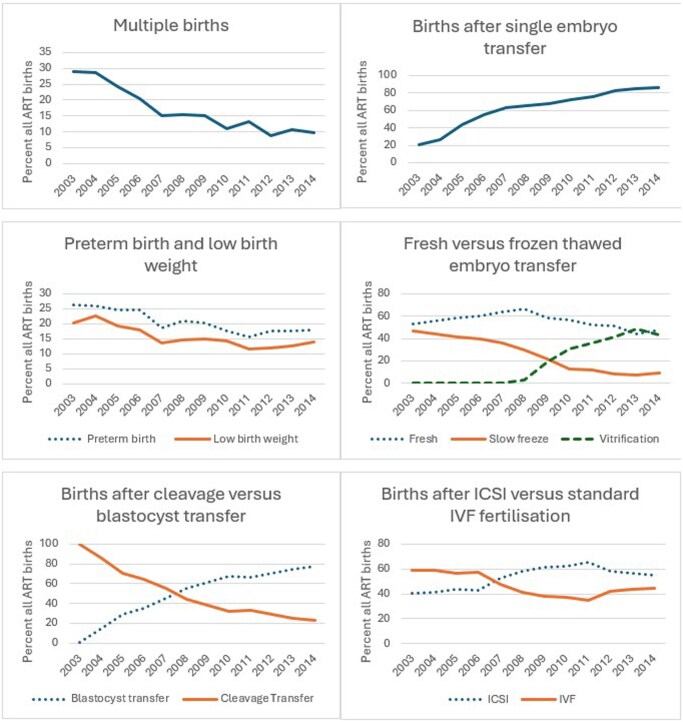
The proportion of multiple births, births after single embryo transfer, preterm births, and children born with low birth weight, births after fresh versus frozen-thawed embryo transfer, births after cleavage versus blastocyst transfer, and births after ICSI versus standard IVF fertilization in all ART-conceived livebirths in Western Australia from 2003 to 2014.

The final cohort comprised 331 290 LB to 208 629 women: 321 931 singletons, 9157 twins, and 202 higher-order multiples. There were 10 126 LB after ART (3.1%), 4102 after OI (1.2%), 11 554 to subfertile untreated women (3.5%), and 305 508 (92.2%) to fertile women. Multiples were more common after ART (14.7%; 1435 twins and 57 triplets) and OI (9.8%; 385 twins, 12 triplets, and 4 quadruplets) than in subfertile untreated (2.8%) and fertile births (2.3%). Multiple births declined over time for women using ART (Fig. 1) and to a lesser extent OI, and remained stable for subfertile untreated and fertile women (Supplementary Fig. S1). For ART births this coincided with an increase in babies born following SET (from 20% of ART births in 2003 to 86% in 2014) and a decline in babies born preterm and with low birth weight. Other changes in the ART group over the study period included an increase in births following ICSI, a rapid rise in births following blastocyst transfer (from 0% to 77% of ART births by 2014); and a decline in fresh embryo transfer following the introduction of rapid embryo freezing (vitrification) from 2008 (Fig. 1).

Parental and birth characteristics associated with deliveries in each conception group are shown in Supplementary Table S1. The mean age of mothers and fathers using ART was 5 years older than in the fertile group, while subfertile untreated and OI mothers and fathers were 3 and 1 year older, respectively. ART mothers were more often primiparous, least likely to smoke in pregnancy, and most likely to have private health insurance. Women in the subfertile treated and untreated groups were more likely to be married or co-habiting and to have Caucasian ethnicity than women in the fertile group. Pre-existing maternal medical conditions were most likely in the subfertile untreated group, whereas complications of pregnancy were most common in the ART group. When we restricted our sample to women with a previous birth, all subfertile groups were more likely to have had a prior preterm birth or stillbirth than the fertile group. Women using ART were most likely to deliver by caesarean section (and emergency caesarean) whether pregnant with a singleton or multiples. ART births were also most likely to be born preterm and low birth weight.

### Prevalence of CP

There were 678 children described as having CP in WA, giving a population prevalence of 2.0/1000 LB (95% CI 1.90–2.21) for the period April 2003 to December 2014. Overall, there was a 5.1% average annual decline in the prevalence of CP during this period (95% CI −6.6% to −3.7%; *P* < 0.001). In the ART group, 29 children had CP (2.9/1000 LB; 95% CI 1.92–4.11), while 16 children with CP were born after OI (3.9/1000 LB; 95% CI 2.23–6.33). There were 23 children with CP in the subfertile untreated group (2.0/1000 LB; 95% CI 1.26–2.99) and 610 children in the fertile group (2.0/1000 LB; 95% CI 1.84–2.16).

There was no increased risk of CP for births to subfertile untreated women, but risk was increased in ART births (fully adjusted RR (aRR) 1.68, 95% CI 1.14–2.48), and highest in OI births (aRR 2.10, 95% CI 1.28–3.44) (Table 1).

**Table 1.** Birth prevalence and risk ratios of cerebral palsy by conception type^1^ (fertile natural conceptions are the reference group).

				Adjusted risk ratios (95% CI)		
	Number CP/total births	Birth prevalence per 1000 live births (95% CI)	Crude risk ratio (95% CI)	All baseline characteristics^2^	Plus maternal pre-existing conditions^3^	Plus adverse obstetric history^4^
**Whole group (including higher order multiples)**						
Fertile NC	610/305 508	2.00 (1.84–2.16)	1.0	1.0	1.0	1.0
Subfertile untreated	23/11 554	1.99 (1.26–2.99)	1.00 (0.66–1.51)	1.15 (0.75–1.74)	1.10 (0.72–1.67)	1.07 (0.70–1.63)
OI	16/4102	3.90 (2.23–6.33)	1.95 (1.19–3.21)	2.22 (1.35–3.64)	2.13 (1.30–3.50)	2.10 (1.28–3.44)
ART	29/10 126	2.86 (1.92–4.11)	1.43 (0.99–2.08)	1.75 (1.18–2.58)	1.72 (1.16–2.53)	1.68 (1.14–2.48)
**All singletons**						
Fertile NC	566/298 370	1.90 (1.74–2.06)	1.0	1.0	1.0	1.0
Subfertile untreated	22/11 226	1.96 (1.23–2.97)	1.03 (0.68–1.58)	1.18 (0.77–1.82)	1.13 (0.74–1.74)	1.11 (0.72–1.71)
OI	13/3701	3.51 (1.87–6.00)	1.85 (1.07–3.21)	2.13 (1.22–3.69)	2.05 (1.18–3.56)	2.02 (1.16–3.51)
ART	11/8634	1.27 (0.64–2.28)	0.67 (0.37–1.22)	0.81 (0.45–1.49)	0.80 (0.44–1.46)	0.78 (0.43–1.43)
**TERM singletons**						
Fertile NC	370/280 569	1.32 (1.19–1.46)	1.0	1.0	1.0	1.0
Subfertile untreated	14/10 346	1.35 (0.74–2.27)	1.03 (0.60–1.75)	1.12 (0.66–1.92)	1.09 (0.64–1.85)	1.08 (0.64–1.85)
OI	5/3405	1.47 (0.48–3.42)	1.11 (0.46–2.69)	1.22 (0.51–2.93)	1.18 (0.49–2.85)	1.18 (0.49–2.85)
ART	7/7718	0.91 (0.36–1.87)	0.69 (0.33–1.45)	0.77 (0.36–1.64)	0.76 (0.36–1.62)	0.76 (0.36–1.62)
**Preterm singletons <37 weeks**						
Fertile NC	196/17 801	11.01 (9.53–12.65)	1.0	1.0	1.0	1.0
Subfertile untreated	8/880	9.09 (3.93–17.83)	0.83 (0.41–1.67)	0.96 (0.47–1.96)	0.95 (0.46–1.96)	0.94 (0.45–1.94)
OI	8/296	27.03 (11.74–52.56)	2.45 (1.22–4.93)	2.89 (1.42–5.87)	2.88 (1.42–5.87)	2.87 (1.41–5.84)
ART	4/916	4.37 (1.19–11.14)	0.40 (0.15–1.06)	0.55 (0.21–1.49)	0.56 (0.21–1.50)	0.56 (0.21–1.50)
**All twins**						
Fertile NC	40/7015	5.70 (4.08–7.76)	1.0	1.0	1.0	1.0
Subfertile untreated	1/322	3.11 (0.08–17.18)	0.54 (0.08–3.95)	0.68 (0.10–4.84)	0.67 (0.10–4.72)	0.71 (0.10–5.01)
OI	3/385	7.79 (1.61–22.60)	1.37 (0.42–4.40)	1.37 (0.43–4.37)	1.17 (0.39–3.48)	1.15 (0.38–3.42)
ART	18/1435	12.54 (7.45–19.75)	2.20 (1.26–3.83)	2.93 (1.47–5.82)	2.92 (1.46–5.84)	2.79 (1.40–5.56)
**TERM twins**						
Fertile NC	5/2881	1.74 (0.56–4.05)	1.0			
Subfertile untreated	0/123	0 (0–29.55)	–			
OI	0/160	0 (0–22.79)	–			
ART	0/427	0 (0–8.60)	–			
**Preterm twins <37 weeks**						
Fertile NC	35/4134	8.47 (5.90–11.76)	1.0	1.0	1.0	1.0
Subfertile untreated	1/199	5.03 (0.13–27.68)	0.59 (0.08–4.31)	0.71 (0.10–5.05)	0.74 (0.10–5.36)	0.78 (0.11–5.68)
OI	3/225	13.33 (2.76–38.47)	1.58 (0.49–5.08)	1.49 (0.46–4.79)	1.16 (0.38–3.56)	1.17 (0.38–3.55)
ART	18/1008	17.86 (10.62–28.08)	2.11 (1.20–3.71)	2.69 (1.38–5.24)	2.82 (1.45–5.49)	2.76 (1.42–5.39)

1 Fertile natural conception (NC), Subfertile untreated, Ovulation Induction as a sole therapy (OI), ART.

2 Baseline characteristics include baby’s year of birth group, maternal age group, parity group, sex, ethnicity, insurance status at birth, marital status, smoking in pregnancy.

3 Pre-existing maternal medical conditions include diabetes, essential hypertension, epilepsy, anxiety and/or depression, cervical surgery.

4 Adverse obstetric history includes prior preterm birth and/or prior stillbirth.

When stratified by plurality and gestational age, the increased risk of CP was restricted to OI singletons (aRR 2.87, 95% CI 1.41–5.84) born preterm (n = 8/296 < 37 weeks), and ART twins (aRR 2.76, 95% CI 1.42–5.39) born preterm (n = 18/1008 < 37 weeks). Additional adjustment for gestational age (in weeks) as a continuous variable had little impact on our RR estimate for preterm ART versus fertile twins (aRR 2.76 changed to 2.64 (95% CI 1.34–5.19)), and slightly more impact for preterm OI versus fertile singletons (aRR 2.87 changed to 2.22 (95% CI 1.07–4.60)) (data not shown). There was no increased risk of CP for any of the subfertile treated or untreated groups in term born singletons or twins.

The pattern of CP risk among conception groups was not altered when infant deaths were excluded, nor was there evidence of correlation within sibships when data were analysed using GEE, with RR estimates largely unchanged (data not shown). The small amount of missing data also had minimal impact on the RRs (Supplementary Table S2).

Considering two time periods (2003–2008 and 2009–2014), the prevalence of CP declined for all conception groups in the second period (Supplementary Table S3). When stratified by plurality, there was no decline in the prevalence of CP for OI singletons (3.50/1000; 95% CI 1.41–7.21) to 3.52/1000 LB; 95% CI 1.29–7.65) or ART twins (12.24/1000; 95% CI 5.61–23.12 to 12.86/1000 LB; 95% CI 5.90–24.27) over time.

### Birth and clinical characteristics of children with CP

A much greater proportion of ART-conceived children with CP were multiples (62.1%) compared with the other conception groups (OI (18.8%), subfertile untreated (4.3%) and fertile (7.2%)) (Table 2). There were more complications of pregnancy, emergency caesarean delivery, preterm birth, and low birthweight in OI and ART births with CP. When stratified by plurality (data not shown), this was no longer the case in ART singletons with CP but still evident for OI singletons. For example, 61.5% of OI singletons with CP were born preterm compared with 35–36% of singletons with CP in the other groups; and 31% weighed <1500 g compared with 9–14% of the other groups.

**Table 2.** Characteristics of women and their children diagnosed with cerebral palsy (CP) by conception group.

	Fertile CP	Subfertile CP	OI CP	ART CP	
	N (%)	N (%)	N (%)	N (%)	*P*-value
Total infants with CP	610	23	16	29	
**Plurality**					
Multiple	44 (7.2)	1 (4.3)	3 (18.8)	18 (62.1)	<0.001
Singleton	566 (92.8)	22 (95.7)	13 (81.3)	11 (37.9)	
Maternal age (years), mean (SD)	29.4 (5.8)	34.3 (5.6)	30.6 (3.3)	34.1 (3.9)	<0.001
Paternal age (years), mean (SD)	32.4 (6.6)	36.0 (6.8)	32.9 (6.6)	35.4 (3.8)	0.007
**Parity**					
Primiparous	270 (44.4)	10 (43.5)	9 (56.2)	17 (63.0)	0.222
Multiparous	338 (55.6)	13 (56.5)	7 (43.8)	10 (37.0)	
**Ethnicity**					
Caucasian	525 (86.3)	21 (91.3)	15 (93.8)	27 (100)	0.129
Other	83 (13.7)	2 (8.7)	1 (6.3)	0	
**Marital** **status**					
Married/co-habiting	502 (83.0)	23 (100)	15 (93.8)	27 (100)	0.004
Other	103 (17.0)	0	1 (6.3)	0	
Missing^1^	3				
**Private health insurance**					
Yes	157 (26.2)	12 (52.2)	12 (75.0)	17 (63.0)	<0.001
No	443 (73.8)	11 (47.8)	4 (25.0)	10 (37.0)	
Missing	8	0	0	0	
**Smoked during pregnancy**					
Yes	109 (17.9)	4 (17.4)	2 (12.5)	2 (7.4)	0.564
No	499 (82.1)	19 (82.6)	14 (87.5)	25 (92.6)	
**Pre-existing maternal medical condition** ^2^					
Yes	200 (32.9)	8 (34.8)	5 (31.3)	7 (25.9)	0.889
No	408 (67.1)	15 (65.2)	11 (68.8)	20 (74.1)	
**Prior stillbirth or preterm birth (where parity > 0)**					
Yes	53 (15.7)	0	1 (14.3)	3 (30.0)	0.201
No	285 (84.3)	13 (100.0)	6 (85.7)	7 (70.0)	
**Complication of pregnancy** ^3^					
Yes	217 (35.7)	9 (39.1)	12 (75.0)	16 (59.3)	0.001
No	391 (64.3)	14 (60.9)	4 (25.0)	11 (40.7)	
**Mode of delivery**					
Emergency caesarean	186 (30.5)	8 (34.8)	7 (43.8)	14 (48.3)	0.140
Elective caesarean	78 (12.8)	6 (26.1)	2 (12.5)	3 (10.3)	
Vaginal	346 (56.7)	9 (39.1)	7 (43.8)	12 (41.4)	
**Sex**					
Female	252 (41.3)	13 (56.5)	10 (62.5)	12 (41.4)	0.184
Male	358 (58.7)	10 (43.5)	6 (37.5)	17 (58.6)	
**Gestational age**					
Preterm <37 w	235 (38.5)	9 (39.1)	11 (68.8)	22 (75.9)	<0.001
Term ≥37 w	375 (61.5)	14 (60.9)	5 (31.3)	7 (24.1)	
**Birth weight**					
<1500 g	103 (16.9)	3 (13.0)	4 (25.0)	11 (37.9)	0.002
1500–2499 g	109 (17.9)	7 (30.4)	5 (31.3)	9 (31.0)	
>2499 g	398 (65.2)	13 (56.5)	7 (43.8)	9 (31.0)	
**Major congenital anomaly**					
Yes	179 (29.3)	5 (21.7)	3 (18.8)	10 (34.5)	0.642
No	431 (70.7)	18 (78.3)	13 (81.3)	19 (65.5)	
**CP characteristics**					
**Motor type**					
Spastic hemiplegia	183 (30.0)	10 (43.5)	2 (12.5)	9 (31.0)	0.885
Spastic diplegia	273 (44.8)	10 (43.5)	11 (68.8)	15 (51.7)	
Spastic quadriplegia	57 (9.3)	1 (4.3)	1 (6.3)	1 (3.4)	
Dyskinesia	56 (9.2)	1 (4.3)	1 (6.3)	2 (6.9)	
Ataxia	26 (4.3)	1 (4.3)	1 (6.3)	2 (6.9)	
Hypotonia	15 (2.5)	0	0	0	
**Gross motor severity**					
Moderate-severe	261 (42.8)	8 (34.8)	6 (37.5)	11 (37.9)	0.809
Minimal-mild	349 (57.2)	15 (65.2)	10 (62.5)	18 (62.1)	
**Epilepsy**					
Yes	161 (26.9)	5 (22.7)	5 (33.3)	3 (10.7)	0.218
No	438 (73.1)	17 (77.3)	10 (66.7)	25 (89.3)	
Missing	11	1	1	1	
**Intellectual impairment**					
Some	261 (43.3)	7 (30.4)	5 (31.3)	6 (21.4)	0.064
None	342 (56.7)	16 (69.6)	11 (68.8)	22 (78.6)	
Missing^1^	7			1	
**Vision impairment**					
Some	154 (26.2)	4 (19.0)	7 (43.8)	6 (21.4)	0.356
None	433 (73.8)	17 (81.0)	9 (56.3)	22 (78.6)	
Missing^1^	23	2		1	
**Hearing impairment**					
Some	43 (7.4)	2 (9.1)	1 (6.3)	2 (7.1)	0.934
None	538 (92.6)	20 (90.9)	15 (93.8)	26 (92.9)	
Missing^1^	29	1		1	
**Speech impairment**					
Some	373 (64.5)	13 (61.9)	7 (43.8)	10 (37.0)	0.012
None	205 (35.5)	8 (38.1)	9 (56.3)	17 (63.0)	
Missing^1^	32	2		2	

1 Missing data are excluded when calculating and comparing proportions across groups.

2 Pre-existing maternal medical conditions include diabetes, essential hypertension, epilepsy, anxiety and/or depression, cervical surgery, thyroid disorder.

3 Complications of pregnancy include threatened abortion, APH, cerclage, vanishing twin survivor, gestational diabetes, preeclampsia, placenta praevia, placental abruption, morbidly adherent placenta, amniotic sac infection, genitourinary infection, vasa praevia, gestational hypertension. Multiple births are not included as a complication of pregnancy but are shown separately.

OI, ovulation induction.

Note: 3 sets of twins both have CP, and 2 of 3 babies in a triplet have CP therefore total numbers for variables differ depending on whether data are based on mother versus child.

In general, clinical characteristics of children with CP were similar across conception groups. ART (51.7%) and particularly OI (68.8%) births appeared to more often present with spastic diplegia as the predominant motor type compared with fertile (44.8%) and subfertile untreated (43.5%) groups.

### OI medications

Most OI births were conceived using clomiphene citrate (n = 3304, 80.5%) and 14 children were diagnosed with CP (4.24/1000; 95% CI 2.32–7.10). Gonadotrophins accounted for almost 15% of OI births (n = 611) and 2 children diagnosed with CP (3.27/1000; 95% CI 0.40–11.77). The remaining 5% of OI births were conceived using either off-label letrozole (n = 123) or tamoxifen (n = 64).

### ART cycle details

The prevalence of CP increased with increasing numbers of embryos transferred, even among singleton births (Table 3). We observed 290 live born twins (4 with CP) and 9 triplets (none with CP) after the transfer of a *single* embryo. Twins after SET comprised an increasing proportion of all ART twins over time (10.2% in the period 2003–2008, 30.7% in the period 2009–2014) and of all ART twins with CP (0 in first period, 44% in the second period) (data not shown). Most twins after SET were same sex twins (n = 263, 90.7%); with the majority conceived after the transfer of a single blastocyst (n = 216; 82.1%). Although numbers are small and differences non-significant, we observed a higher prevalence of CP after the use of donor oocytes/embryos compared with a woman’s own oocytes/embryos (7.2 vs 2.7/1000 LB).

**Table 3.** Prevalence of cerebral palsy (CP) by number of embryos transferred and own versus donor oocytes/embryos.

	ART no CP N (%)	ART CP N (%)	Prevalence/1000 live births (95% CI)	Crude RR^1^ (95% CI)	Adjusted RR^2^ (95% CI)
Total N	10 097	29	2.86 (1.92–4.11)		
Number embryos transferred—all births					
1	6881 (68.1)	10 (34.5)	1.45 (0.70–2.67)	1	1
2	3151 (31.2)	18 (62.1)	5.68 (3.37–8.96)	3.91 (1.81–8.47)	4.44 (1.58–12.53)
3+	65 (0.6)	1 (3.4)	15.15 (0.38–81.55)	10.44 (1.36–80.40)	13.42 (1.45–123.94)
Number embryos transferred—singletons					
1	6586 (76.4)	6 (54.5)	0.91 (0.33–1.98)	1	1
2	2005 (23.3)	4 (36.4)	1.99 (0.54–5.09)	2.19 (0.62–7.74)	2.55 (0.56–11.63)
3+	32 (0.4)	1 (9.1)	30.30 (0.77–157.59)	33.29 (4.12–268.95)	39.17 (2.87–533.71)
Number of embryos transferred—twins					
1	286 (20.2)	4 (22.2)	13.79 (3.77–34.94)	1.10 (0.37–3.33)	1.13 (0.20–6.58)
2	1107 (78.1)	14 (77.8)	12.49 (6.84–20.87)	1	1
3+	24 (1.7)	0	–	–	
Own oocytes or embryos	9823 (97.3)	27 (93.1)	2.74 (1.81–3.99)	1	1
Donor oocytes or embryos	274 (2.7)	2 (6.9)	7.25 (0.88–25.93)	2.64 (0.63–11.06)	3.09 (0.74–12.82)

1 Risk ratio.

2 Adjusted for fresh versus frozen-thawed embryo transfer, baby year of birth (1993–2008, 2009–2014), ICSI versus IVF, blastocyst versus cleavage stage transfer (and SET vs other for donor/own analysis).

Table 4 shows the prevalence of CP by different ART cycle characteristics for singletons born following SET and twins following DET. These comparisons were based on small numbers of children with CP, reflected in the wide CIs around risk estimates. However, adjusted risk ratios were doubled following blastocyst versus cleavage-stage transfer for both singletons and twins, and the transfer of fresh rather than frozen-thawed embryos and standard IVF versus ICSI fertilization also seemed to confer higher risks for twins. The prevalence of CP was lower in the second time period for both singletons following SET and twins following DET.

**Table 4.** Prevalence of cerebral palsy (CP) by different ART cycle characteristics for singletons following single embryo transfer (SET) and twins following double embryo transfer (DET).

	ART no CP	ART CP	Prevalence/1000 live births (95% CI)	Crude RR^1^ (95% CI)	Adjusted RR^2^ (95% CI)
**Singletons after SET** ^3^ **-Total N**	6586	6	0.91 (0.33–1.98)		
IVF	2939 (44.6)	2 (33.3)	0.68 (0.08–2.45)	1	1
ICSI	3647 (55.4)	4 (66.7)	1.10 (0.30–2.80)	1.61 (0.30–8.79)	1.71 (0.31–9.43)
Fresh ET	3589 (54.5)	3 (50.0)	0.84 (0.17–2.44)	1	1
Slow freeze	988 (15.0)	1 (16.7)	1.01 (0.03–5.62)	1.21 (0.13–11.63)	1.29 (0.20–8.39)
Vitrification	2009 (30.5)	2 (33.3)	0.99 (0.12–3.59)	1.19 (0.20–7.12)	1.22 (0.199–8.39)
Cleavage	1740 (26.4)	1 (16.7)	0.57 (0.01–3.20)	1	1
Blastocyst	4846 (73.6)	5 (83.3)	1.03 (0.33–2.40)	1.79 (0.21–15.35)	2.09 (0.36–12.02)
2003–2008	1779 (27.0)	2 (33.3)	1.12 (0.14–4.05)	1	1
2009–2014	4807 (73.0)	4 (66.7)	0.83 (0.23–2.13)	0.74 (0.14–4.04)	0.60 (0.11–3.28)
**Twins after DET-Total N**	1107	14	12.49 (6.84–20.87)		
IVF	526 (47.5)	9 (64.3)	16.82 (7.72–31.69)	1	1
ICSI	581 (52.5)	5 (35.7)	8.53 (2.78–19.80)	0.51 (0.17–1.50)	0.52 (0.18–1.51)
Fresh ET	632	11	17.11 (8.57–30.40)	1	1
Frozen^4^	475	3	6.28 (1.30–18.23)	0.37 (0.10–1.31)	0.33 (0.09–1.25)
Cleavage	828	8	9.57 (4.14–18.77)	1	1
Blastocyst	279	6	21.05 (7.76–45.26)	2.20 (0.77–6.29)	2.60 (0.93–7.32)
2003–2008	627	9	14.15 (6.49–26.69)	1	1
2009–2014	480	5	10.31 (3.36–23.89)	0.73 (0.25–2.16)	0.72 (0.24–2.17)

1 Risk ratio.

2 Model adjusted for all other factors in the table.

3 Multiples after SET are not shown in this table including 290 twins (4 with CP) and 9 triplets (0 with CP).

4 Slow-freeze and vitrification combined to allow model to converge (includes 348 slow freeze (3 with CP), 128 vitrification (0 with CP), 2 fresh+slow-freeze (0 with CP)).

### Monozygotic twinning

Supplementary Table S4 shows the estimated monozygotic twinning rate per 100 deliveries across our four conception groups. These data indicate an almost 3-fold increase in monozygotic twinning for ART births compared with fertile (and other) conception groups and a greater risk following blastocyst versus cleavage stage transfer (whether SET or DET). Just over 2% of deliveries after SET were twin deliveries and we estimate that 81% of these were monozygous. The monozygotic twinning rate after blastocyst-SET was 2.0% versus 1.2% after cleavage-SET.

## Discussion

This population-based study compared the risk of CP across four conception groups: births to fertile women who conceived naturally (reference group), women with indicators of subfertility who conceived naturally, and women who had used fertility treatments (OI medications or ART) to conceive. We hypothesized, based on our previous work examining pre-existing maternal medical conditions, pregnancy complications, and perinatal outcomes across these groups (Hansen *et al.*, 2024), that the risk of CP would increase with subfertility and from less invasive (OI) to more invasive (ART) treatments. Instead, we found that there was no increased risk of CP in children born to untreated subfertile women, the risk of CP had declined in ART-conceived births since our previous study spanning birth years 1994–2002 (Goldsmith *et al.*, 2018), and risk was highest (doubled) for births to women who had used OI medications as a sole therapy. Clinical outcomes were broadly similar between children with CP from the different conception groups, although OI-conceived singletons with CP were much more likely to be born preterm, and were more often described with spastic diplegia (69%) than the other groups (45%).

### ART-conceived births

We observed an average annual 5% decline in the prevalence of CP for all WA births over the study period. Declining prevalence of pre/perinatal CP has also been reported for the whole of Australia (Smithers-Sheedy *et al.*, 2024) and in other high-income countries (McIntyre *et al.*, 2022). The prevalence of CP in births to women who conceived naturally declined from 2.5/1000 LB in our previous study (Goldsmith *et al.*, 2018) (birth years 1994–2002) to 2.0/1000 LB in this cohort (birth years 2003–2014). The prevalence of CP in ART-conceived births declined much more from 7.2/1000 LB to 2.9/1000 LB resulting in a reduction in relative risk of CP over time. Similar declines have been reported in the Nordic countries (Spangmose *et al.*, 2021; Carlsen *et al.*, 2023) coinciding, as in WA, with a strong shift to SET, leading to fewer multiple and preterm births. SET has long been recognized as a way of reducing CP prevalence following ART (Stromberg *et al.*, 2002; Hvidtjorn *et al.*, 2010). In our earlier study (Goldsmith *et al.*, 2018) only 6% of births were conceived following the transfer of a single embryo, compared with 68% in this study (and 86% of ART births in 2014). We anticipate that the association with CP will decline further, as SET continues to increase in Australia (94.9% in 2023) (Kotevski *et al.*, 2025), along with an ongoing decline in the background prevalence of pre/perinatal CP (Australian Cerebral Palsy Register, 2025).

We observed a large decline in the prevalence of CP in ART singletons (from 5.2/1000 LB in our previous study to 1.3/1000) and the risk of CP was no longer increased compared with naturally conceived singletons, again comparable with Nordic data from similar time periods (Spangmose *et al.*, 2021; Carlsen *et al.*, 2023). In contrast to the decline we observed for ART singletons, we found an increase in CP prevalence for ART twins from 9.4/1000 LB in our previous study (1994–2002) (Goldsmith *et al.*, 2018) to 12.5/1000 LB in the current study. The prevalence of CP declined in naturally conceived twins in WA over the same period from 8.4/1000 to 5.7/1000 LB, consistent with declines in CP prevalence reported for all twins in Australia and Europe (Perra *et al.*, 2021; Sellier *et al.*, 2021). These opposing trends resulted in an almost 3-fold increased risk of CP in ART-conceived twins not reported in previous studies (Wang *et al.*, 2021; Cavero-Ibiricu *et al.*, 2024), with risk confined to preterm births. Factors contributing to this risk are difficult to determine with our data but may relate to patients with poorer prognosis opting for the transfer of two embryos and/or changes in ART clinical practice in WA such as the strong shift to blastocyst transfer.

Practice guidelines issued by the European Society for Human Reproduction and Embryology state that no clinical or embryological factor justifies the recommendation of DET over SET (Alteri *et al.*, 2024). However, they concede that patients who present with several poor prognostic factors (advanced age, poor-quality embryos, no LB from previous cycles) may be offered DET. The guidelines also recommend that blastocysts should only be transferred in a SET because of the high risk of multiple pregnancy and complications after the transfer of two blastocysts (Chen *et al.*, 2020; Hill *et al.*, 2020; Zhu *et al.*, 2020a), and their higher monozygotic twin potential (Hviid *et al.*, 2018). In agreement with these data, we observed: (i) that women who gave birth following DET were older than those who conceived following SET (median age 36 vs 34 years) (data not shown); (ii) a higher twinning rate after the transfer of two blastocysts (26/100 deliveries) versus two cleavage-stage embryos (21/100 deliveries); and (iii) a higher prevalence of CP in twins born after blastocyst versus cleavage-stage DET (21.0/1000 LB vs 9.6/1000).

Twins after SET may result from intercourse around the time of embryo transfer or from embryo splitting (Hviid *et al.*, 2018; Zhaffal *et al.*, 2024). With the shift to SET, there has been growing interest in the higher incidence of monozygotic twinning after ART (Hviid *et al.*, 2018; Busnelli *et al.*, 2019; Kadam *et al.*, 2023; Paul *et al.*, 2025) with blastocyst transfer identified as an important risk factor (Busnelli *et al.*, 2019; Paul *et al.*, 2025). Indeed, we estimated that the monozygotic twinning rate after blastocyst-SET in our study was 2.0% versus 1.2% after cleavage-SET (and 0.5% after fertile natural conception). Risks associated with multiple pregnancies are compounded in monozygotic twinning by complications such as higher rates of preterm birth and low birthweight (Rissanen *et al.*, 2022), growth discordance (Lewi *et al.*, 2014); and twin-to-twin transfusion syndrome (Hviid *et al.*, 2018), all associated with greater risk of CP (Boghossian *et al.*, 2019; Yan *et al.*, 2023). Twins after SET comprised an increasing proportion of all ART twins over time in our study, and of all ART twins with CP. Most twins after SET were same-sex twins (90.7%) and we estimated that 81% were monozygous, with the majority conceived after the transfer of a single blastocyst. Blastocyst-stage transfer was used much more frequently in WA than in the Nordic countries during this period (57% vs 11% (Spangmose *et al.*, 2021)) and may account for some of the higher risk of CP in ART-conceived twins in our study.

Treatment strategies to reduce monozygotic twinning after SET should be explored and data from a recent large Australian study suggest that this could include the transfer of a vitrified-thawed rather than fresh blastocyst (Paul *et al.*, 2025). When stratified by fresh versus frozen-thawed embryo transfer, the odds of monozygotic twinning were greatest after the transfer of a fresh blastocyst versus cleavage stage embryo (aOR 2.2 (95% CI 1.8–2.6)), and lowest (and non-significant) after the transfer of a vitrified-thawed blastocyst versus cleavage stage embryo (aOR 1.2 (95% CI 0.8–1.9)). Potential mechanisms linking blastocyst transfer to monozygotic twinning include the extended time in culture media that may lead to hardening of the zona pellucida, destabilize intracellular bonds, or alter cellular signalling resulting in greater susceptibility for splitting of the inner cell mass (Busnelli *et al.*, 2019; Chen *et al.*, 2023; Zhao *et al.*, 2025). Vitrification may protect against these structural alterations, or the freezing/thawing process may act as a selection mechanism, allowing only higher-quality, more resilient embryos to survive, reducing the likelihood of splitting due to developmental stress. Finally, fresh blastocyst transfers occur shortly after ovarian stimulation into a uterus with altered hormonal profiles which may disrupt endometrial receptivity or alter implantation processes increasing the likelihood of splitting (Mateizel *et al.*, 2016; Liu *et al.*, 2020; Paul *et al.*, 2025).

We observed an increase in risk of CP with increasing number of embryos transferred for all ART births and for ART singletons. We compared CP prevalence by ART technique for singletons following SET and twins following DET to remove, as far as possible, any effects of vanishing twins and splitting embryos. Despite the small sample size, births after blastocyst transfer consistently showed an increased prevalence of CP compared with births after cleavage-stage transfer. This warrants scrutiny in larger, more recent cohorts since the shift to blastocyst transfer has continued in Australia; 93.3% of embryo transfer cycles in 2023 (Kotevski *et al.*, 2025).

### OI-conceived births

This is the first population-based study to report on the risk of CP in births conceived after OI in Australia, finding risk remained doubled after adjusting for a broad range of potential confounders. Risk was largely confined to OI singletons born preterm and remained after adjusting for gestational age (in weeks). Our findings are similar to those reported in a Finnish study (Klemetti *et al.*, 2010), whereas a Danish study (Hvidtjorn *et al.*, 2010) found no increased risk when stratified by plurality.

OI is generally the first-line treatment for women with absent or infrequent ovulation caused by PCOS. These women have higher rates of obesity, diabetes, hypertensive, and endocrine disorders, experience more pregnancy complications such as preeclampsia and gestational diabetes, and their infants are at increased risk of preterm birth and low Apgar scores (Doherty *et al.*, 2015; Teede *et al.*, 2023) all of which may contribute to an increased risk of CP. The increased prevalence of multiples after OI also contributes to the risk of CP; 10% of OI births and 19% of OI-conceived children with CP were multiples. Multiple pregnancy following OI may also have contributed to the increased risk of CP in preterm singletons if they were more likely exposed to vanishing twins. While SET may prevent most of the excess burden of CP associated with ART by reducing multiple pregnancies and singletons exposed to a vanishing twin, controlling the number of developing oocytes following OI is far more difficult (Klemetti *et al.*, 2010; Bergh *et al.*, 2020). However, there may be room to improve clinical practice through milder OI strategies and increased ultrasound monitoring with cycle cancelation if several leading follicles are observed (Bergh *et al.*, 2020; Moore *et al.*, 2022; Teede *et al.*, 2023). Indeed, in a prospective observational study of anovulatory women undergoing OI with gonadotrophins using a low-dose step-up protocol, a multiple pregnancy rate of 2% was reported without compromising the cumulative live birth rate (Pontré *et al.*, 2020). This low multiple pregnancy rate was achieved through careful monitoring and cycle cancelation in the case of excessive or no follicular response (10.5% cycles).

Most OI births (80.5%) were conceived using clomiphene citrate (CC) during our study period, however, off-label use of letrozole for OI has increased with international guidelines recommending it should be the first-line OI treatment for women with anovulatory PCOS (Teede *et al.*, 2023). Recent reviews suggest it improves monofollicular development (Abu-Zaid *et al.*, 2024), pregnancy, and live birth rates (Franik *et al.*, 2022; Teede *et al.*, 2023; Abu-Zaid *et al.*, 2024) compared with CC. While there were no children diagnosed with CP in the 123 births conceived with letrozole, much larger cohorts are required to assess the safety of this medication.

### Subfertile natural conceptions

We included a subfertile comparison group to attempt to disentangle the association between subfertility, use of fertility treatment, and risk of CP. Similar to Zhu *et al.* (2010), we found that despite subfertile women having slightly higher risks of complications (e.g. threatened miscarriage, preterm birth (Hansen *et al.*, 2024)), this did not translate to an increased risk of CP.

### Strengths and limitations

The strengths of this large study include linkage of statutory, whole-population datasets enabling us to identify different methods of conception and to examine associated risks of CP using data from one of the longest standing CP registers worldwide (Bower *et al.*, 2015). Small amounts of missing data for two covariates did not appear to materially affect our RR estimates. We acknowledge several limitations: (i) identification of our OI group was reliant on medication dispensing rather than consumption (Hansen *et al.*, 2024); (ii) small numbers of births with CP in the subfertile groups resulted in imprecise effect estimates for some subgroup analyses and prevented more detailed analyses of pregnancy complications, ART cycle characteristics and their association with CP; (iii) we accounted for a range of pre-existing medical conditions and adverse obstetric history but were unable to adjust for maternal BMI (only available for the last three birth years in our cohort); (iv) as zygosity data were not available, we estimated the rate of monozygotic twinning using Weinberg’s formula which may have lead to some misclassification; and finally, (v) more recent data are needed to understand CP risk associated with current clinical practice.

This study is also limited by our inclusion of only non-Aboriginal women. The much greater discrepancy observed between the proportion of Aboriginal births in the fertile (6.5%) versus ART group (0.5%), compared with other ethnic groups in our data, is highly unlikely to be due to a lower need for fertility treatment services (Gilbert *et al.*, 2021). Rather, there is likely inequitable access and barriers to fertility services for Aboriginal women, particularly for those living outside urban areas, which requires further investigation in dedicated research together with Aboriginal researchers (Gilbert *et al.*, 2020, 2021; Smithers-Sheedy *et al.*, 2024).

## Conclusions

ART-conceived births now comprise 6.4% of Australian births (Australian Institute of Health and Welfare, 2025; Kotevski *et al.*, 2025) highlighting the importance of understanding health outcomes and safe treatment practices. This study confirms an important decline in the risk of CP for ART births in Australia. Consistent with similar reports from the Nordic countries, this decline coincides with the strong shift to SET and reductions in preterm and multiple births. While SET does not eliminate the risk of multiple pregnancy, it is currently the best strategy to lower multiple pregnancy rates and risk of CP following ART. Future, larger studies should investigate in more detail the suggestion in our study that CP risk may be increased following blastocyst transfer and in twins after SET. Data about OI (as a sole therapy) are not routinely collected in most countries including Australia, but OI-conceived births had the highest risk of CP and may warrant closer scrutiny. Pre-treatment counselling for couples seeking ART should continue to promote SET while incorporating the small risk of embryo splitting, and risk of multiple birth should be discussed with those planning to use OI.

## Supplementary Material

deag076_Supplementary_Figure_S1

deag076_Supplementary_Table_S1

deag076_Supplementary_Table_S2

deag076_Supplementary_Table_S3

deag076_Supplementary_Table_S4

## Data Availability

The data that support the findings of this study are not publicly available due to privacy and ethical restrictions.
