# Supplementary material for: Fertility treatment and risk of cerebral palsy: has the association changed in Australia?
Source: Hum Reprod. 2026 May 24;41(7):1183–96. doi: 10.1093/humrep/deag076 (PMC13334919; doi:10.1093/humrep/deag076)
Supplement: deag076_Supplementary_Figure_S1 [file deag076_supplementary_figure_s1.pdf]

Human Reproduction, 2026, 1–1
https://doi.org/10.1093/humrep/deag076
Supplementary Data

human reproduction

OXFORD

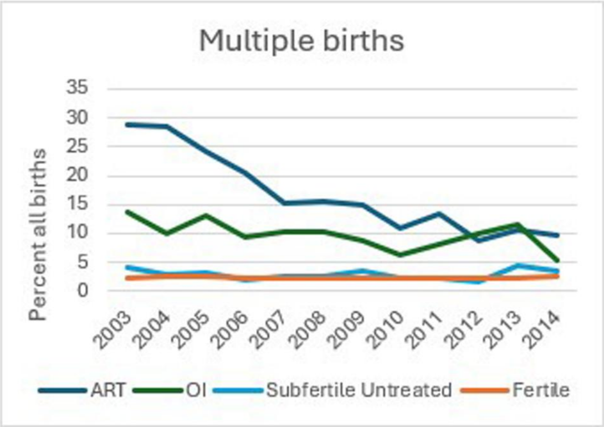

Supplementary Figure S1. The proportion of multiple births after ART, ovulation induction, untreated subfertility, and natural conception to fertile women in Western Australia from 2003 to 2014.
