# Supplementary material for: Fertility treatment and risk of cerebral palsy: has the association changed in Australia?
Source: Hum Reprod. 2026 May 24;41(7):1183–96. doi: 10.1093/humrep/deag076 (PMC13334919; doi:10.1093/humrep/deag076)
Supplement: deag076_Supplementary_Table_S1 [file deag076_supplementary_table_s1.pdf]

Human Reproduction, 2026, 1–1
https://doi.org/10.1093/humrep/deag076
Supplementary Data

human reproduction

OXFORD

Supplementary Table S1. Characteristics of women and their babies by conception group.

	Singletons					Multiples				
	Fertile NC, N (%)	Subfertile, N (%)	OI, N (%)	ART, N (%)	*P*-value	Fertile NC, N (%)	Subfertile, N (%)	OI, N (%)	ART, N (%)	*P*-value
Total infants	298 370 (92.7)	11 226 (3.5)	3701 (1.1)	8634 (2.7)		7138 (76.3)	328 (3.5)	401 (4.3)	1492 (15.9)	
Maternal age (years), mean (SD)	29.6 (5.5)	33.1 (4.8)	31.4 (4.6)	34.7 (4.4)	<0.001	30.3 (5.3)	33.8 (4.8)	31.1 (4.0)	34.9 (4.5)	<0.001
median	30	33	31	35		31	34	31	35	
Paternal age (years), mean (SD)	32.4 (6.4)	35.5 (5.7)	33.8 (5.4)	37.7 (6.2)	<0.001	32.9 (6.2)	36.0 (5.6)	33.3 (5.1)	37.8 (6.2)	<0.001
Median	32	35	33	37		33	36	33	37	
**Parity**										
Primiparous	127 074 (42.6)	3659 (32.6)	1991 (53.8)	5390 (62.4)	<0.001	1331 (37.0)	57 (34.5)	106 (53.3)	471 (63.0)	<0.001
Multiparous	171 296 (57.4)	7567 (67.4)	1710 (46.2)	3244 (37.6)		2267 (63.0)	108 (65.5)	93 (46.7)	277 (37.0)	
**Ethnicity**										
Caucasian	244 433 (81.9)	9998 (89.1)	3344 (90.4)	7626 (88.3)	<0.001	2979 (82.8)	141 (85.5)	180 (90.5)	654 (87.4)	<0.001
Other	53 937 (18.1)	1228 (10.9)	357 (9.6)	1008 (11.7)		619 (17.2)	24 (14.5)	19 (9.5)	94 (12.6)	
**Marital status**										
Married/co-habiting	264 379 (88.6)	10 691 (95.2)	3582 (96.8)	8266 (95.7)	<0.001	3132 (87.0)	155 (93.9)	188 (94.5)	724 (96.8)	<0.001
Other	31 484 (10.6)	474 (4.2)	104 (2.8)	321 (3.7)		444 (12.3)	10 (6.1)	10 (5.0)	22 (2.9)	
Missing[1]	2507 (0.8)	61 (0.5)	15 (0.4)	47 (0.5)		22 (0.6)	0	1 (0.5)	2 (0.3)	
**Private health insurance**										
Yes	108 266 (36.3)	7213 (64.3)	2487 (67.2)	6665 (77.2)	<0.001	1281 (35.6)	116 (70.3)	134 (67.3)	553 (73.9)	<0.001
No	186 747 (62.6)	3941 (35.1)	1193 (32.2)	1907 (22.1)		2309 (64.2)	48 (29.1)	65 (32.7)	194 (25.9)	
Missing[1]	3357 (1.1)	72 (0.6)	21 (0.6)	62 (0.7)		8 (0.2)	1 (0.6)	0	1 (0.1)	
**Smoked during pregnancy**										
Yes	37 200 (12.5)	760 (6.8)	179 (4.8)	215 (2.5)	<0.001	495 (13.8)	11 (6.7)	7 (3.5)	24 (3.2)	<0.001
No	261 170 (87.5)	10 466 (93.2)	3522 (95.2)	8419 (97.5)		3103 (86.2)	154 (93.3)	192 (96.5)	724 (96.8)	
**Pre-existing maternal medical condition**[2]										
Yes	65 260 (21.9)	3127 (27.9)	906 (24.5)	2013 (23.3)	<0.001	895 (24.9)	52 (31.5)	52 (26.1)	150 (20.1)	0.005
No	233 110 (78.1)	8099 (72.1)	2795 (75.5)	6621 (76.7)		2703 (75.1)	113 (68.5)	147 (73.9)	598 (79.9)	
**Prior stillbirth or preterm birth** (where parity > 0)										
Yes	11 454 (6.7)	801 (10.6)	152 (8.9)	390 (12.0)	<0.001	173 (7.6)	7 (6.5)	12 (12.9)	38 (13.7)	0.002
No	159 842 (93.3)	6766 (89.4)	1558 (91.1)	2854 (88.0)		2094 (92.4)	101 (93.5)	81 (87.1)	239 (86.3)	
**Complication of pregnancy**[3]										
Yes	66 327 (22.2)	3006 (26.8)	1081 (29.2)	2941 (34.1)	<0.001	1477 (41.1)	64 (38.8)	98 (49.2)	369 (49.3)	<0.001
No	232 043 (77.8)	8220 (73.2)	2620 (70.8)	5693 (65.9)		2121 (58.9)	101 (61.2)	101 (50.8)	379 (50.7)	
**Mode of delivery**										
Emergency	44 487 (14.9)	1713 (15.3)	648 (17.5)	1829 (21.2)	<0.001	2215 (31.0)	103 (31.4)	113 (28.2)	573 (38.4)	<0.001
Elective	51 896 (17.4)	3442 (30.7)	892 (24.1)	2703 (31.3)		2316 (32.4)	157 (47.9)	191 (47.6)	647 (43.4)	
Vaginal	201 987 (67.7)	6071 (54.1)	2161 (58.4)	4102 (47.5)		2607 (36.5)	68 (20.7)	97 (24.2)	272 (18.2)	
**Sex**										
Female	145 754 (48.9)	5455 (48.6)	1797 (48.6)	4151 (48.1)	0.849	3487 (48.9)	158 (48.2)	193 (48.1)	752 (50.4)	0.696
Male	152 612 (51.1)	5771 (51.4)	1904 (51.4)	4483 (51.9)		3651 (51.1)	170 (51.8)	208 (51.9)	740 (49.6)	
Indeterminate	4									
**Gestational age (weeks)** mean (SD)	38.9 (1.7)	38.4 (1.8)	38.5 (1.9)	38.2 (2.1)	<0.001	35.2 (2.8)	35.4 (2.4)	35.0 (2.9)	34.6 (3.0)	<0.001
Preterm <37 w	17 801 (6.0)	880 (7.8)	296 (8.0)	916 (10.6)		4256 (59.6)	205 (62.5)	241 (60.1)	1065 (71.4)	
Term ≥37 w	280 569 (94.0)	10 346 (92.2)	3405 (92.0)	7718 (89.4)		2882 (40.4)	123 (37.5)	160 (39.9)	427 (28.6)	
**Birth weight (g)**										
mean (SD)	3399 (532)	3360 (534)	3329 (544)	3293 (576)	<0.001	2392 (591)	2410 (542)	2377 (611)	2290 (602)	<0.001
<1500 g	1848 (0.6)	91 (0.8)	41 (1.1)	120 (1.4)		590 (8.3)	21 (6.4)	38 (9.5)	161 (10.8)	
1500–2499 g	10 827 (3.6)	478 (4.3)	143 (3.9)	450 (5.2)		3132 (43.9)	158 (48.2)	171 (42.6)	740 (49.6)	
>2499 g	285 695 (95.8)	10 657 (94.9)	3517 (95.0)	8064 (93.4)		3416 (47.9)	149 (45.4)	192 (47.9)	591 (39.6)	
**Major congenital anomaly**										
Yes	12 765 (4.3)	537 (4.8)	195 (5.3)	563 (6.5)	<0.001	474 (6.6)	29 (8.8)	24 (6.0)	117 (7.8)	0.153
No	285 605 (95.7)	10 689 (95.2)	3506 (94.7)	8071 (93.5)		6664 (93.4)	99 (91.2)	377 (94.0)	1375 (92.2)	

1 Missing data are shown as a proportion of total N for each conception group but are excluded when calculating and comparing proportions across groups.
2 Pre-existing maternal medical conditions include diabetes, essential hypertension, epilepsy, anxiety and/or depression, cervical surgery, thyroid disorder.
3 Complications of pregnancy include threatened abortion, APH, cerclage, vanishing twin survivor, gestational diabetes, preeclampsia, placenta praevia, placental abruption, morbidly adherent placenta, amniotic sac infection, genitourinary infection, vasa praevia, gestational hypertension.
