# Supplementary material for: Fertility treatment and risk of cerebral palsy: has the association changed in Australia?
Source: Hum Reprod. 2026 May 24;41(7):1183–96. doi: 10.1093/humrep/deag076 (PMC13334919; doi:10.1093/humrep/deag076)
Supplement: deag076_Supplementary_Table_S2 [file deag076_supplementary_table_s2.pdf]

Human Reproduction, 2026, 1–1
https://doi.org/10.1093/humrep/deag076
Supplementary Data

human reproduction OXFORD

Supplementary Table S2. Effect of missing data on risk ratios for pre/perinatal cerebral palsy (CP) for ART, ovulation induction, and subfertile untreated births compared with fertile natural conceptions (Fertile NC).

	N as per crude model (no missing data) (all basic covariates available)			N as per fully adjusted model (cases with missing PHI/marital status are excluded)	
	**N**	**Crude RR (95% CI)**	**RR (95% CI) Basic[1]**	**N**	**RR (95% CI) Basic[1]**
**Pre/perinatal CP**					
**Whole group (including HOMs[2])**					
Fertile NC[3]	610/305 508	1.00 (reference)	1.00 (reference)	599/299 618	1.00 (reference)
Subfertile untreated	23/11 554	1.00 (0.66–1.51)	1.01 (0.67–1.54)	23/11 420	1.02 (0.68–1.56)
Ovulation induction	16/4102	1.95 (1.19–3.21)	1.90 (1.15–3.12)	16/4064	1.92 (1.17–3.15)
ART	29/10 126	1.43 (0.99–2.08)	1.48 (1.01–2.18)	29/10 011	1.50 (1.02–2.20)
**Singletons**					
Fertile NC	566/298 370	1.00 (reference)	1.00 (reference)	555/292 539	1.00 (reference)
Subfertile untreated	22/11 226	1.03 (0.68–1.58)	1.04 (0.68–1.59)	22/11 094	1.05 (0.69–1.61)
Ovulation induction	13/3701	1.85 (1.07–3.21)	1.80 (1.04–3.13)	13/3665	1.83 (1.05–3.17)
ART	11/8634	0.67 (0.37–1.22)	0.68 (0.38–1.24)	11/8525	0.69 (0.38–1.25)
**Twins**					
Fertile NC	40/7015	1.00 (reference)	1.00 (reference)	40/6956	1.00 (reference)
Subfertile untreated	1/322	0.54 (0.08–3.95)	0.64 (0.09–4.51)	1/320	0.64 (0.09–4.51)
Ovulation induction	3/385	1.37 (0.42–4.40)	1.18 (0.40–3.55)	3/383	1.18 (0.40–3.55)
ART	18/1435	2.20 (1.26–3.83)	2.81 (1.45–5.46)	18/1429	2.80 (1.44–5.45)

Risk ratio (RR) estimates are calculated using a basic Poisson model adjusted for key covariates[1] with no missing data on the whole dataset and the reduced dataset (where cases with missing private health insurance status and/or marital status are excluded). Differences in RR estimates are solely due to missing data.

1 Basic adjusted Poisson model includes year of birth group, maternal age group, parity group, sex, ethnic origin, smoking, pre-existing diabetes, essential hypertension.

2 HOMS, higher order multiples.
