# Supplementary material for: Fertility treatment and risk of cerebral palsy: has the association changed in Australia?
Source: Hum Reprod. 2026 May 24;41(7):1183–96. doi: 10.1093/humrep/deag076 (PMC13334919; doi:10.1093/humrep/deag076)
Supplement: deag076_Supplementary_Table_S3 [file deag076_supplementary_table_s3.pdf]

Human Reproduction, 2026, 1–1
https://doi.org/10.1093/humrep/deag076
Supplementary Data

human reproduction
OXFORD

Supplementary Table S3. Birth prevalence of cerebral palsy (CP) in Western Australia for each conception group by time period and plurality.

	Fertile Natural Conception		Subfertile Untreated		Ovulation Induction		ART	
	CP/total LB[2]	Birth prevalence CP/1000 LB (95% CI)	CP/total LB	Birth prevalence CP/1000 LB (95% CI)	CP/total LB	Birth prevalence CP/1000 LB (95% CI)	CP/total LB	Birth prevalence CP/1000 LB (95% CI)
**All births**								
2003–2008	318/136 551	2.33 (2.08–2.60)	14/5098	2.75 (1.50–4.60)	10/2244	4.46 (2.14–8.18)	14/3711	3.77 (2.06–6.32)
2009–2014	292/168 957	1.73 (1.54–1.94)	9/6456	1.39 (0.64–2.64)	6/1858	3.23 (1.19–7.02)	15/6415	2.34 (1.31–3.85)
**PR[1] (95% CI)**		**0.74 (0.63–0.87)**		**0.51 (0.22–1.17)**		**0.72 (0.26–1.99)**		**0.62 (0.30–1.28)**
Total period	610/305 508	2.00 (1.84–2.16)	23/11 554	1.99 (1.26–2.99)	16/4102	3.90 (2.23–6.33)	29/10 126	2.86 (1.92–4.11)
**Singletons**								
2003–2008	292/133 322	2.19 (1.95–2.46)	14/4958	2.82 (1.54–4.73)	7/1998	3.50 (1.41–7.21)	5/2943	1.70 (0.55–3.96)
2009–2014	274/165 048	1.66 (1.47–1.87)	8/6268	1.28 (0.55–2.51)	6/1703	3.52 (1.29–7.65)	6/5691	1.05 (0.39–2.29)
**PR[1] (95% CI)**		**0.76 (0.64–0.89)**		**0.45 (0.19–1.08)**		**1.00 (0.34–2.99)**		**0.62 (0.19–2.03)**
Total period	566/298 370	1.90 (1.74–2.06)	22/11 226	1.96 (1.23–2.97)	13/3701	3.51 (1.87–6.00)	11/8634	1.27 (0.64–2.28)
**Twins**								
2003–2008	24/3146	7.63 (4.89–11.33)	0/140	0 (0–26.01)	3/233	12.88 (2.66–37.16)	9/735	12.24 (5.61–23.12)
2009–2014	16/3869	4.14 (2.37–6.71)	1/182	5.49 (0.14–30.23)	0/152	0 (0–23.98)	9/700	12.86 (5.90–24.27)
**PR[1] (95% CI)**		**0.54 (0.29–1.02)**		–		–		**1.05 (0.42–2.63)**
Total period	40/7015	5.70 (4.08–7.76)	1/322	3.11 (0.08–17.18)	3/385	7.79 (1.61–22.60)	18/1435	12.54 (7.45–19.75)

[1] PR, prevalence ratio comparing two time periods.
[2] LB, live births.
