# Supplementary material for: Fertility treatment and risk of cerebral palsy: has the association changed in Australia?
Source: Hum Reprod. 2026 May 24;41(7):1183–96. doi: 10.1093/humrep/deag076 (PMC13334919; doi:10.1093/humrep/deag076)
Supplement: deag076_Supplementary_Table_S4 [file deag076_supplementary_table_s4.pdf]

*Human Reproduction*, 2026, 1–1
https://doi.org/10.1093/humrep/deag076
**Supplementary Data**

human reproduction
OXFORD

**Supplementary Table S4.** Monozygotic twinning (MZT) rates according to method of conception and (for ART births) length of embryo culture.

| Method of conception[1] | Total deliveries[2] | Twin deliveries | Unlike sex twin deliveries | Twin/ 100 deliveries | MZT/100 deliveries[3] | 95% CI MZT/ 100 deliveries | PR[4] (95% CI) |
|---|---|---|---|---|---|---|---|
| Fertile NC | 301 925 | 3555 | 996 | 1.2 | 0.52 | 0.49–0.54 | Reference |
| Subfertile | 11 389 | 163 | 55 | 1.4 | 0.47 | 0.35–0.61 | 0.90 (0.68–1.18) |
| OI | 3895 | 194 | 87 | 5.0 | 0.51 | 0.31–0.79 | 0.99 (0.64–1.54) |
| ART | 9363 | 729 | 296 | 7.8 | 1.46 | 1.23–1.73 | **2.83 (2.38–3.36)** |
| DET | 2576 | 567 | 277 | 22.0 | 0.50 | 0.27–0.86 | Reference |
| SET | 6742 | 150 | 14 | 2.2 | 1.81 | 1.50–2.16 | **3.59 (2.03–6.34)** |
| Cleavage SET | 1768 | 27 | 3 | 1.5 | 1.19 | 0.74–1.81 | Reference |
| Blastocyst SET | 4974 | 123 | 11 | 2.5 | 2.03 | 1.66–2.46 | **1.71 (1.07–2.73)** |
| Cleavage DET | 2020 | 423 | 207 | 20.9 | 0.45 | 0.20–0.84 | Reference |
| Blastocyst DET | 556 | 144 | 70 | 25.9 | 0.72 | 0.20–1.83 | 1.61 (0.50–5.22) |

[1] Fertile natural conception (NC), subfertile untreated, ovulation induction as a sole therapy (OI), ART double embryo transfer (DET), single embryo transfer (SET).
[2] Singletons and twins only.
[3] Estimated using Weinberg's formula (e.g. for Blastocyst SET MZT/100 deliveries: (123−(2×11))/4974 = 2.03).
[4] Prevalence ratio.
